# The “horizontal” components of the real gravity are not relevant to ocean dynamics

**DOI:** 10.1038/s41598-022-09967-3

**Published:** 2022-04-11

**Authors:** Edmund K. M. Chang, Christopher L. P. Wolfe

**Affiliations:** School of Marine and Atmospheric Sciences, Stony Brook University, Stony Brook, USA

**Keywords:** Physical oceanography, Fluid dynamics

**arising from**: P. C. Chu; *Scientific Reports* 10.1038/s41598-021-82882-1 (2021).

## Introduction

In a recent paper^1^, the author derived equations on coordinate surfaces equivalent to oblate spheroids that account for the deviation of geopotential surfaces from spherical surfaces due to the centrifugal force caused by Earth’s rotation (but then approximated them as spheres—see Supplementary Information). The author argued that the true gravity not only has a vertical component in these coordinates, but also a horizontal component due to variations in Earth’s mass distribution. The magnitude of the horizontal component of gravity was claimed to be an order of magnitude larger than the horizontal components of Coriolis force and pressure gradient force that form the main geostrophic balance for large-scale oceanographic flow. The author argued that omission of the horizontal component of gravity is not warranted.

We do not agree with the author that current ocean models (and theories)—and by implication atmospheric ones^2^—err in *omitting* the horizontal component of gravity. Let us start from Newton’s second law of motion for large-scale oceanic flow, taking the Boussinesq approximation as the author did^1^ (note that to avoid confusion we will use the same equations and notations as those used by the author):

1 $${\rho }_{0}\left[\frac{D\mathbf{U}}{Dt}+2{\varvec{\Omega}}\times \mathbf{U}\right]=-\nabla p+\rho \nabla V+{\rho }_{0}\mathbf{F}$$

In (1), *V* is the true potential for the true gravity,

2 $$V=P+{P}_{R}$$

where *P* is the true gravitational potential that includes spatial inhomogeneities, and *P*_*R*_ is the potential associated with the centrifugal force due to the rotation of the earth,

3 $${P}_{R}= \frac{1}{2} {\Omega }^{2}{r}^{2}{\mathrm{cos}}^{2}\varphi$$

Surfaces of constant *V* are close to oblate spheroidal, but with wiggles due to the inhomogeneities in the Earth’s mass distribution.

Ocean modeling requires expressing (1) in a coordinate system. There are many possible choices for doing so. The author chose a coordinate system in which the direction of the vertical is perpendicular to exact oblate spheroids. In such a coordinate system the direction of the true gravity is not parallel to the vertical and hence true gravity has horizontal components.

In contrast, common practice in ocean modeling^3, 4^ is that the vertical is *defined* as the direction opposite to the true gravity ($\nabla V$). That is, in a direction that is perpendicular to the true geopotential surfaces (surfaces of constant *V*). Under this definition, the horizontal component of true gravity is *exactly* zero. In this coordinate system, the vertical is not actually perpendicular to hypothetical spheroidal surfaces as argued by the author but is instead perpendicular to the local total geopotential surfaces of constant *V* which deviate slightly from spheroidal^3^.

We first estimate how different this coordinate is from a coordinate that is exactly spheroidal. The angle, $\delta \alpha$, between the direction of true gravity and the “vertical” on a true spheroid can be estimated by the value of the “horizontal” component of gravity on a spheroid (as defined by the author) divided by the “vertical” component, which, based on fig. 2 of the paper^1^, is $\delta \alpha \lesssim {10}^{-4}$ radians (< 0.01°) in most locations. This angle is very small because true gravity on a spheroid is dominated by its vertical component. Given this small angle, the approximation made in common ocean (and atmospheric) modeling is to approximate this non-spheroidal coordinate system as a spheroidal coordinate system. Note that conceptually, this approximation is different from neglecting the horizontal component of true gravity in a truly spheroidal coordinate system. The actual approximation is to approximate a nearly spheroidal coordinate system, in which the true gravity is exactly vertical, with a spheroidal coordinate system^3^. With this interpretation, the dominant error of the approximation now appears in the metric terms.

Let us estimate how large this error might be. The metric terms arise when the acceleration vector is expressed in a coordinate system that is not globally Cartesian, as follows^5^:

4 $$\frac{D\mathbf{U}}{Dt}=\hat{i}\frac{Du}{Dt}+\hat{j}\frac{Dv}{Dt}+\hat{k}\frac{Dw}{Dt}+u\frac{D\hat{i}}{Dt}+v\frac{D\hat{j}}{Dt}+w\frac{D\hat{k}}{Dt},$$

where *u*, *v*, and *w* are the three velocity components, and $\hat{i}$, $\hat{j}$, and $\hat{k}$ are the three local unit vectors of the coordinate system. The last 3 terms on the RHS of (4) are the metric terms, which arise due to the local unit vectors changing direction following fluid motion. The order of magnitude of the dominant metric terms in (4) can be estimated by ${U}^{2}\delta \alpha /L$, where $U$ is the horizontal velocity scale, $\delta \alpha$ is the change of angle (in radians) of the frame of reference at different spatial locations, and $L$ is the spatial scale over which this angle changes. For the standard metric term, $\delta \alpha \sim 1$ over a spatial scale $L$ equivalent to the radius of the earth $a$. Hence the usual scale of the metric terms in the horizontal momentum Eqs.^5^ is ${U}^{2}/a$. For the deviations of the true geopotential surfaces from spheroidal, the angle is $\delta \alpha \lesssim {10}^{-4}$ radians (< 0.01°) in most locations as estimated above. The spatial scale $L$, again based on Fig. 2 of the paper^1^, is very conservatively estimated as $\gtrsim 100$ km. Using these values gives $\delta \alpha /L<{10}^{-9} {\mathrm{m}}^{-1}$, which is at least 2 orders of magnitude smaller than $\delta \alpha /L$ for the regular metric terms (${a}^{-1} \sim {10}^{-7} {\mathrm{m}}^{-1}$). Hence ignoring the wiggles in the true geopotential surfaces produces errors on the order of < 1% of the metric terms. Even using a velocity scale as large as 1 m s^−1^, the error of omitting these perturbations to the metric terms in the horizontal momentum equations is less than 10^−9^ m s^−2^. This is < 10^−5^ of the author’s estimate of the error, and several orders of magnitude smaller than the horizontal pressure gradient force and Coriolis force. Note that using this velocity scale, the magnitude of the horizontal Coriolis force is 10^−4^ m s^−2^. This estimate confirms that the errors made by approximating the near oblate spheroidal coordinate in which the true gravity is exactly vertical with a truly oblate spheroidal coordinate system is negligible, as suggested in ocean dynamics texts^3, 4^.

Physically, for a static fluid, the pressure field exactly compensates for the total gravity field to produce a net force of zero throughout the fluid. In this hypothetical static state, the pressure surfaces and the true geopotential surfaces are perfectly aligned. If we use a coordinate system that is not exactly aligned with the true geopotential surfaces, such as the perfect spheroid used by the author, even in the static case, there will be horizontal gravity forces, but these will be *exactly* cancelled by the static horizontal pressure gradient force. Balanced motion involves force balance that deviates from this static balance, with the perturbation in pressure gradient force balancing the Coriolis and other forces such as viscosity. Because the horizontal gravity is still balanced by the static pressure gradient force, it does not enter into the dynamical force balance. In such a coordinate system, dynamic balance appears to be a small residual from the dominant horizontal static balance, just like what happens in the author’s analysis and equations. By defining the vertical to be perpendicular to the local true geopotential surfaces rather than perfect spheroids, the static “horizontal” component of gravity and the counteracting pressure gradient force are now absorbed into the vertical direction, and the true dynamical balance becomes the dominant horizontal balance. The cost of doing this is adding less than a 1% error to the metric terms as estimated above.

Note that if we start with exactly spherical coordinates, gravity has strong horizontal components due to the centrifugal force and the non-spherical mass distribution of the earth. We redefine (or tilt) the vertical to be opposite to the sum of gravitational force and centrifugal force, and in this new (nearly spheroidal) coordinate system, the horizontal component of gravity becomes exactly zero and does not enter the horizonal dynamic equations. This nearly spheroidal coordinate is then approximated as a spherical coordinate—an approximation that the author also took^1^. In this coordinate, the horizontal components of gravity (including those of the centrifugal force) that appear in a truly spherical coordinate do not appear in the equations, since these “horizontal” components have been absorbed into the vertical by the definition of the vertical. It’s ironic that the author appears to accept this redefinition of the vertical to eliminate the horizontal components of gravity related to the centrifugal force and near spheroidal mass distribution of the earth (see Supplementary Information for a discussion of the errors made by this approximation—these errors are again small and not of leading order) but does not accept the further refinement of the vertical to account for the “horizontal” components of the gravitational force associated with the small deviations of the geopotential surfaces from spheroidal.

Further, it is standard oceanographic practice to report measurements as functions of pressure, *p*, rather than depth or height. In this coordinate system, the mean sea surface is always near $p=0$ regardless of the shape of the geoid because the mean sea surface lies on a surface of constant true geopotential. In pressure coordinates, the hydrostatic equation in fact becomes an equation for the true geopotential as a function of pressure and it is gradients of this true geopotential on pressure surfaces that drives horizontal motion. As discussed above, since any “horizontal” component of gravity is exactly balanced by a static horizontal pressure gradient force, the gradient of the true geopotential on pressure surfaces is free of the “horizontal” component of gravity, regardless of the coordinate system used.

## Supplementary Information

Supplementary Information.

## Data Availability

No data were used apart from those found in the references cited.
