# Supplementary material for: The “horizontal” components of the real gravity are not relevant to ocean dynamics
Source: Sci Rep. 2022 Apr 11;12:6027. doi: 10.1038/s41598-022-09967-3 (PMC9001721; doi:10.1038/s41598-022-09967-3)
Supplement: Supplementary file 1 — Supplementary Information. [file 41598_2022_9967_MOESM1_ESM.pdf]

Supplementary Information for

The "horizontal" components of the real gravity are not relevant to ocean dynamics

Edmund K.M. Chang[*1] and Christopher L.P. Wolfe[1]

[1]School of Marine and Atmospheric Sciences, Stony Brook University

[*]Corresponding Author: Edmund K.M. Chang

Email: kar.chang@stonybrook.edu

Supplementary Information

One can compare the error of ignoring the "wiggles" in the true geopotential surfaces (i.e., approximating them as spheroidal) to that of approximating spheroidal surfaces (due to the action of the centrifugal force) as exactly spherical – an approximation that the author also made[1]. In the latter case, there are again errors in the metric terms, and in this case, $\delta\alpha$ is the angle between the "vertical" as defined as perpendicular to spherical versus spheroidal surfaces. This angle $\delta\alpha$ can be estimated by the horizontal component of the centrifugal force divided by gravity, which has a maximum value of $\delta\alpha \sim (\Omega^2 a)/2g \sim 10^{-3}$. Here the scale L is the radius of the earth a, hence $\delta\alpha/L \sim 10^{-10}\ \mathrm{m}^{-1}$ and is slightly smaller than that for the error due to ignoring the wiggles in the true geopotential surfaces as estimated in the main text. Note that while the horizontal component of the centrifugal force is stronger than the "horizontal" component of gravity associated with the wiggles in the true geopotential surfaces, the scale over which the centrifugal force varies is larger, hence the error associated with ignoring its variations can be smaller. However, this approximation also introduces small errors into the horizontal derivatives ($\sim 10^{-3}$) because distances between longitudes and latitudes are slightly different on spheroids and spheres[6]. In spherical coordinates, the value of gravity should additionally be approximated as independent of latitude to avoid unphysical sources of vorticity[7]. Numerical experiments[7] showed that while the errors may be visible in a 10-day forecast, they are certainly not of leading order. In addition, errors associated with approximating the acceleration due to gravity as independent of latitude dominate over geometric errors, so the errors associated with approximating spheroidal surfaces as spherical are likely larger than the geometric errors for approximating the true geopotentials as spheroids. Note that the latter approximation introduces negligible errors into the value of gravity.

References:

6. Veronis, G. Large Scale Ocean Circulation. *Adv. Appl. Mech.*, **13**, 1-92, doi:10.1016/S0065-2156(08)70143-1. https://www.sciencedirect.com/science/article/pii/S0065215608701431 (1973)

7. Benard, P. An assessment of global forecast errors due to the spherical geopotential approximation in the shallow-water case. *Q. J. R. Meteorol. Soc.*, **141**, 195-206 (2015).
